# Propionic Acid: Method of Production, Current State and Perspectives

**DOI:** 10.17113/ftb.58.02.20.6356

**Published:** 2020-06

**Authors:** Vahid Ranaei, Zahra Pilevar, Amin Mousavi Khaneghah, Hedayat Hosseini

**Affiliations:** 1 Department of Public Health, School of Public Health, Hamadan University of Medical Sciences, Hamadan, Iran; 2 Student Research Committee, Department of Food Sciences and Technology Department, National Nutrition and Food Technology Research Institute, Faculty of Nutrition Sciences and Food Technology, Shahid Beheshti University of Medical Sciences, Tehran 1981619573, Iran; 3 Department of Food Science, Faculty of Food Engineering, State University of Campinas (UNICAMP), São Paulo, Brazil; 4 Department of Food Sciences and Technology Department, National Nutrition and Food Technology Research Institute, Faculty of Nutrition Sciences and Food Technology, Shahid Beheshti University of Medical Sciences, Tehran 1981619573, Iran; 5 Food Safety Research Center, Shahid Beheshti University of Medical Sciences, Tehran, Iran

**Keywords:** propionic acid, *Propionibacterium freudenreichii*, *Propionibacterium acidipropionici*, glycerol fermentation

## Abstract

During the past years, there has been a growing interest in the bioproduction of propionic acid by *Propionibacterium*. One of the major limitations of the existing models lies in their low productivity yield. Hence, many strategies have been proposed in order to circumvent this obstacle. This article provides a comprehensive synthesis and review of important biotechnological aspects of propionic acid production as a common ingredient in food and biotechnology industries. We first discuss some of the most important production processes, mainly focusing on biological production. Then, we provide a summary of important propionic acid producers, including *Propionibacterium freudenreichii* and *Propionibacterium acidipropionici*, as well as a wide range of reported growth/production media. Furthermore, we describe bioprocess variables that can have impact on the production yield. Finally, we propose methods for the extraction and analysis of propionic acid and put forward strategies for overcoming the limitations of competitive microbial production from the economical point of view. Several factors influence the propionic acid concentration and productivity such as culture conditions, type and bioreactor scale; however, the pH value and temperature are the most important ones. Given that there are many reports about propionic acid production from glucose, whey permeate, glycerol, lactic acid, hemicelluloses, hydrolyzed corn meal, lactose, sugarcane molasses and enzymatically hydrolyzed whole wheat flour, only few review articles evaluate biotechnological aspects, *i.e*. bioprocess variables.

## INTRODUCTION

Among all industrially available organic acids, propionic acid (PA) and its derivatives can be mentioned as important chemical intermediates, which are mostly used in a variety of industrial applications as antimicrobial agents for a broad spectrum of microorganisms (*1*, *2*), anti-inflammatory substance, exhibiting analgesic and antipyretic properties (*3*, *4*), herbicides, controlling both monocotyledonous and dicotyledonous plants (*5*, *6*), preservatives in bakery and cheese products (*7*, *8*), artificial flavours and fragrances (*9*), pharmaceuticals (*10*), precursors of cellulose acetate propionate (CAP) (*11*), *etc.*

Propionic acid or ethanecarboxylic acid is one of the top 30 potential biomass candidates as determined by the US Department of Energy (DOE) (*12*). The annual world market for propionic acid was 350 000 tonnes (*13*), which was approximately equal to 770 million pounds in 2006 (*14*). The largest and fastest growing markets are Europe and Asia Pacific, respectively. The highest revenue share (in %) belongs to agriculture, food and beverage, personal care and pharmaceutical sectors. The world market demand for PA was 38 and 400 kilotonnes in 2007 and 2013, respectively. It is expected to reach 470 in 2020 (1.53 billion US$) (*6*).

The term ’propionic’ derives from the Greek words ’protos’ (first) and ’pion’ (fat) and was first discovered by Johann Gottlieb in 1844 as a result of the conversion of pyruvate into PA *via* succinate decarboxylation or acrylate pathways (*15*).

Glycerol as a by-product from biodiesel production receives a great attention as a carbon source for the production of propionic acid (*16*). However, there are other cheap carbon sources such as glucose, lactose, lactic acid, hemicelluloses, whey permeate (*17*), hydrolyzed corn meal (*18*), sugarcane molasses (*19*) and enzymatically hydrolyzed whole wheat flour (*20*). Lactic acid and carbohydrates from biomass can be chemically transformed into propionic acid by using Zn as a reducing agent and Co as a catalyst with strong activity (*21*).

Although expensive, anaerobic fluidized (*22*), plant (*23*) and multi-point fibrous (*24*) bed reactors (*e.g.* cotton fibres) (*25*), calcium alginate (*26*) and calcium polygalacturonate beads (*27*) and expanded bed adsorption (*28*) and granular sludge bed (*17*) reactors have been proposed for propionic acid production by *Propionibacterium freudenreichii*. In this review paper, the most critical aspects of PA including its chemical properties, microbial production utilizing both immobilized and free bacteria in recombinant and wild forms, consuming various sources of carbon and nitrogen, the effect of controlled culture systems and its industrial applications are reviewed.

### Microbial production of propionic acid

Propionic acid is fermented by *Propionibacterium freudenreichii* ssp. shermanii (*26*), *Selenomonas ruminantium* (*29*), *Propionibacterium acidipropionici* (*30*), *Propionibacterium jensenii* (*31*), *Propionibacterium thoenii* (*32*), *Veillonella gazogenes* (*33*), *Veillonella criceti* (*34*), *Veillonella alcalescens* (*35*), *Veillonella parvula* (*36*), *Megasphaera elsdenii* (*37*), *Clostridium homopropionicum* (*36*), *Bacteroides* spp. and *Fusobacterium necrophorum* (*38*). Propionic acid can also be a by-product of biological fermentation for vitamin B12 (dimethyl benzimidazole as a precursor) (*39*), trehalose (from levulinic acid) (*40*) and porphyrin (by δ-aminolevulinic acid and porphobilinogen) production (*41*).

Larger amounts of volatile fatty acids are produced by gut microbiota through anaerobic fermentation of dietary fibre, non-volatile fatty acids and proteins (*42*). Dietary fibre, as the primary substrate of colon microbiota, is metabolized to pyruvate, which is converted to PA (*43*). Undigested carbohydrates in small intestine are fermented to propionic, butyric and acetic acids, and gases including H_2_, CO_2_ and CH_4_ are released, together with heat due to exothermic reaction (*44*). Formation of volatile fatty acids in the intestine depends on different extrinsic and intrinsic factors regarding environmental conditions, substrate availability (*e.g.* carbon limitation) and bacterial species (*45*).

In biosynthesis of propionic acid from glycerol, *P. acidipropionici* has shown higher efficiency in terms of conversion yield and fermentation time than other strains such as *Propionibacterium acnes* and *Clostridium propionicum* (*22*, *46*). Mutation of *P. acidipropionici* has lead to the increase of H^+^-ATPase expression and resistance to pH changes (*14*). However, it should be considered that high propionic acid concentration causes carboxylate inhibition during fermentation. Excess propionic acid can be excluded by using extractive fermentation. Low acid concentration ensures higher product yield and lower amounts of by-products (*37*). For propionic acid extraction, only undissociated acids are drawn out by hexane solution as the solvent (*47*). To find a solution to the major problem of organic acid production, acid recovery of ten solvents was examined. Alcohols and 1-butanol were considered as the best recovery solution and cost-effective extractor, respectively (*48*).

Supercritical carbon dioxide (solvent) and tri-*n*-octylamine (reactant) with high pressure (16 MPa) can be applied for PA extraction from aqueous solutions at low temperature (35 °C). These methods with 94.7% extraction efficiency are superior to the physical extraction of organic acids (*49*).

Recovery of propionic acid by electrodialysis from cell-free fermentation medium leads to higher product concentration (*50*). As opposed to aerobic fermentation, anaerobic fermentation is difficult to monitor, which could be overcome by measuring the oxidoreduction potential as an easy and cost-effective method (*51*). Interconversion of NADH/NAD^+^ redox pair can be used for regulation of propionic acid production through oxidoreduction potential control (*52*).

Besides environmental pollution from fossil resources, irreversible fuels should be substituted as their prices get higher due to depletion of petroleum (*53*) and necessity of specific catalysts (*54*). However, industrial production of propionic acid by fermentation cannot be feasible unless process cost is eqiuvalent to the production of a PA by petrochemical routes such as ethylene carbonylation, hydrocarbons and propanol oxidation (*55*, *56*). The production of PA from industrial wastes such as glycerol or molasses makes biomass-based PA economically competitive to fossil-based PA (*22*, *57*).

## PROCESS PARAMETERS INFLUENCING MICROBIAL PRODUCTION AND PRODUCTIVITY OF PROPIONIC ACID

Fermentation of propionic acid encounters some limitations such as inhibition of cell growth during the process (*25*) and formation of organic acids. Among 17 strains of *Propionibacterium*, *Propionibacterium acidipropionici* AT CC 4875 has been reported to achieve highest propionic acid yield (*58*).

The presence of KCl in glycerol medium improves the production of trehalose by *Propionibacterium freudenreichii,* sensitive to osmotic stress (*59*). Although corn mash medium increases product yield, this medium reduces productivity when it is used without cyanocobalamin (*58*). *Megasphaera elsdenii* prefers lactic acid (lactate) to glucose despite the pre-growth on glucose medium. *M. elsdenii* converts lactic acid into monocarboxylic volatile fatty acids as C2-5 acids (*60*).

Organic acids including *n*-propanol and acetic, formic and succinic acids are formed as the by-products of propionic acid production from glycerol (*46*). Gases such as CO_2_ from glucose (*14*) or lactose (*61*) are also limiting factors that are produced by *P. acidipropionici,* but all the by-products can be significantly reduced by extractive fermentation with hollow fibre membrane as extractor and amine as the extracting chemical (*62*, *63*).

Higher temperatures during fermentation lead to a higher quantity of propionic than acetic acid due to degradation of volatile fatty acids (>C3) (*64*). Electrodialysis in conjunction with ultrafiltration can exhibit higher volumetric productivity when used for the production of organic acids (*65*). The same results are achieved in the chemically based production of propionic acid in electrocatalytic membrane reactors through oxidation of propanol (*56*). The self-renewable embedding of propionibacteria in calcium alginate and calcium polygalacturonate gels is hard to achieve (*23*). A xylan hydrogel matrix for immobilization of *Propionibacterium acidipropionici* has shown the productivity of 0.88 g/(L·h) during continuous fermentation in stirred tank. This approach is associated with high cell adhesion to solid carrier surfaces even at high dilution rates, resulting in 99.7 g/L of dry cell density (*66*). Spin filters (with 5 μm pore size) can be applied for *in situ* cell retention to achieve fourfold productivity of propionic acid through the continuous fermentation (0.9 g/(L·h)) compared to common batch fermentation (*67*).

### Choice of microorganism

Propionibacteria are pleomorphic catalase- and Gram-positive, anaerobic, aerotolerant bacteria that produce propionic acid as the main product *via* fermentation by Wood-Werkman cycle (*68*). There are two main pathways for the fermentation of PA from pyruvate: *via* decarboxylation of succinate or conversion of acrylate with lactate (precursor) (*69*). Three biotin-dependent carboxylases have shown to control carbon flux through the dicarboxylic acid pathway in the cycle. Their combination with glucose and glycerol as carbon sources results in increased acid concentration and higher productivity (*70*). Productivity can also be improved by the application of metabolic engineering, for which *Escherichia coli* is the most widely used host (*71*). Phosphoenolpyruvate carboxylase enzyme from *Escherichia coli* has been cloned into *Propionibacterium freudenreichii*. Higher propionic acid yield was produced at a faster rate by a mutant strain of *Propionibacterium freudenreichii* than by the wild-type (*72*). High propionate concentration has been achieved through fermentation of glycerol by *E. coli,* which is comparable to anaerobic fermentation by *Propionibacterium* (*73*).

*Veillonella criceti* as a Gram-negative bacterium can convert lactate to propionate with high productivity rate of 39 g/(L·h) (*74*). *Bacillus coagulans* and *Lactobacillus zeae* are able to convert glucose or other carbon sources to lactate (*74*, *75*). The mutant strain of *Bacillus coagulans* has shown high final titre (145 g/L), yield (0.98 g/g) and d-lactate purity (99.9%) (*76*). To avoid product and substrate inhibition, PA (product) and lactate (substrate) should be removed from fermentor and kept at low concentrations (*74*).

### Control of the pH during fermentation

Fermentation encounters feedback inhibition *via* propionic acid. This event can be controlled by different methods including choosing acid/propionate-tolerant strains, pH control by the inclusion of buffers or bases and pH adjustment and shift control strategies (*37*, *77*, *78*).

At constant pH, lactate exhibits higher product yield than glucose and lactose and limits succinic acid production. Moreover, pH control is easier when using lactate as a carbon source in immobilized cell bioreactors (continuous type) (*61*).

The production of propionic acid can be improved by controlling pH during fermentation (*57*, *79*). Since the optimum pH for growth of *Propionibacterium* is higher than for *Clostridium*, a pH shift from 6 to 8 leads to a higher proportion of propionic than butyric acid from glucose medium (*80*). In Swiss-type cheese, reducing lactose and higher pH values (5.20-5.35) leads to acceleration of PA fermentation (*81*).

The acid-tolerant mutant strain of *Propionibacterium acidipropionici* has been physiologically (*82*) and molecularly (*55*) studied by genome shuffling and proteomics, respectively. Understanding the details of acid tolerance mechanisms and factors contributing to changes in acid accumulation may lead to an increase in propionic acid production by regulation of the fermentation process (*83*).

Results of serial studies demonstrate that genome shuffling can be used to produce the mutant by inactivated protoplast fusion, and acid-tolerant mutant bacteria are affected by proton pump of the membrane, glutamate decarboxylase and arginine deaminase (*82*). The pH change caused by the production of acid metabolites affects the membrane and cell wall structures (*73*). Therefore, the effect of pH is an important issue in fermentation process due to the high sensibility of biological materials. Many studies have been performed to find optimum pH for the growth of *Propionibacterium*. By using the strategy of pH adjustment in two stages (pH maintained at 6.5 for 48 h and then at 6.0), it was possible to increase PA yield significantly (from 14.58 to 19.21 g/L) compared to the production at constant pH=6.0 (*23*).

The type of substrate is another parameter that influences propionic acid yield since its conversion ratio can be directly affected. Depending on the type of substrate, the pH control might become harder to manage. It was stated that lactate, as carbon basis, presented some advantages compared to glycerol and sugarcane molasses (*84*). It was noticed that when glycerol and sugarcane molasses were used, faster pH variation was observed; however, it was slow when lactate was used (*19*).

### Temperature

Temperature is an important factor in all fermentation processes that affects overall process yield by directly influencing biochemical performance. Many genera of *Propionibacterium* have been studied, and each genus requires different optimum temperature. In the oldest available literature, the optimum temperature was determined in the range 14–40 °C (*85*). In the following studies, the optimum temperature for PA production was recorded mostly between 30 and 40 °C (*86*, *87*).

### Carbon source

Many different types of carbon sources as the substrate can be considered as the most expensive conventional raw materials in the fermentation process (Table 1 (*11*, *14*, *23*, *24*, *30*, *58*, *74*, *88*-*96*)).

**Table 1:** Yield, productivity and final titer of propionic acid production under different experimental conditions

**Strain**	**Carbon source**				**Temperature/°C**	**pH**	**Yield/(g/g)**		**Productivity/(g/(L·h))**	**Final titer/(g/L)**		**Reference**
***Propionibacterium acidipropionici* ACK-Tet (mutant of ATCC 4875)**	Glucose				32	6.5	0.54		0.41	97		(*14*)
***Propionibacterium acidipropionici* ATCC 4875**	Mature Jerusalem artichoke tubercle roots (40 g/L fructose and 20 g/L glucose)				32	6.5	0.42		3.69	22.9		(*88*)
***Propionibacterium acidipropionici* ATCC 4875**	Glucose				32	6.5	0.45		2	45		(*58*)
***Propionibacterium acidipropionici* F3E8**	Glucose				32	7.0	0.55		0.84	40		(*125*)
***Propionibacterium acidipropionici* ATCC 55737**	Glucose				32	7.0	0.42		0.62	27		(*125*)
***Propionibacterium acidipropionici* ATCC 4875**	Glucose				32	7.0	0.45		0.61	30		(*125*)
***Propionibacterium acidipropionici* CGMCC 1.2232 (*Propionibacterium acidipropionici* ATCC 4875)**	Whey lactose				32	6.0	0.45		0.2	27		(*90*)
***Propionibacterium freudenreichii* CCTCC M207015**	Glucose				35	5.5–7.0	-		0.12	14.58		(*24*)
***Propionibacterium acidipropionici* DSM 4900**	Glycerol				30	6.5	0.74		0.29	20		(*91*)
***Propionibacterium freudenreichii* CCTCC M207015**	Glucose				35	6.0	-		0.16	34.03		(*23*)
***Propionibacterium acidipropionici* ATCC4965**	Glucose/Glycerol				30	6.5	0.57		0.152	21.9		(*89*)
***Propionibacterium acidipropionici* ATCC4965**	Glucose				30	6.5	0.30		0.068	11.5		(*89*)
***Propionibacterium acidipropionici* ATCC4965**	Glycerol				30	6.5	0.47		0.108	18.1		(*89*)
***Propionibacterium acidipropionici* ACT-1 (adapted from ATCC 4875)**	Glucose	32	5.5	0.52	0.162			52.1			(*11*)	
***Propionibacterium acidipropionici* ACT-1 (adapted from ATCC 4875)**	Glucose	32	5.5	0.62	0.159			42.7			(*11*)	
***Propionibacterium acidipropionici* ACT-1 (adapted from ATCC 4875)**	Soy molasses	32	6.5	0.39	0.35			54.1			(*11*)	
***Propionibacterium acidipropionici* ATCC 4875**	Glucose	32	6.5	0.43	2.23			55.7			(*126*)	
***Propionibacterium acidipropionici* CGMCC 1.2230**	Glycerol	30	7.0	0.57	0.19			48			(*30*)	
***Propionibacter freudenrechii* ssp. *shermanii* PTCC 1661**	Glycerol	30	6.5-7.0	0.724	0.113			-			(*127*)	
***Propionibacter freudenrechii* T82**	Pure sugars	37	6.5	0.30	0.039			7.66			(*128*)	
***Propionibacter freudenrechii* T82**	Pure sugars	30	6.5	0.32	0.043			7.66			(*128*)	
***Propionibacterium freudenreichii* CICC 10019**	Glucose	30	7.0	0.66	0.33			85.4			(*129*)	
***Propionibacterium freudenreichii* CICC 10019**	Crop stalk hydrolysates	30	7.0	0.75	0.35			91.4			(*129*)	
***Bacillus coagulans* DSMZ 2314 and *Veillonella criceti*** **DSMZ 20734**	Glucose	37	6.2	0.35	0.63			-			(*74*)	

To maintain high productivity and reduce the cost of production, many studies have been carried out to evaluate the possible use of cheap renewable agro-industrial sources and wastes (*i.e.* sugarcane molasses and glycerol) (*9*, *26*). The use of cheap substrates including corn gluten, corn steep liquor, sulphite and wood pulp waste liquors, lignocelluloses, flour hydrolysates and whey as carbon sources (*88*) can be an alternative for a more viable product.

*Propionibacterium* is capable of consuming various sources of carbon such as glucose (*89*), fructose (*16*), sucrose (*9*), lactose (*90*), glycerol (*91*) and molasses (*86*). Different productivity and conversion yield can be achieved dependent on the type of applied carbon source. Propionic acid productivity based on glycerol (*46*), hemicellulose (*97*) and glycerol/lactate (*19*) as carbon sources is 0.18, 0.28 and 0.113 g/(L·h), respectively. Contrary to lactic acid and glucose, higher glycerol concentration results in increased productivity and lower conversion yield (*46*). Glycerol feed with the constant rate of 0.01 L/h (72-120 h) has led to the maximum PA production by *P. acidipropionici* that can be scaled up to industrial level (*30*). The productivity of PA is higher in fermentation of glycerol than in fermentation of glucose. In contrast, the increase in glucose/glycerol mass ratio increases the vitamin B12 productivity (*98*).

By application of vegetable oil, biodiesel industry provides a great amount of glycerol as a by-product, which can be considered as an economically viable feedstock for industrial production of PA (*23*). Glycerol can be used in PA fermentation as a carbon source (*26*, *89*). Although it is an excellent reducing agent, which favours the production of PA (*99*), it might lead to redox imbalance in the metabolism that might affect the cell evolution and lower the yield when used as the sole carbon source in fermentation. Besides low price and availability of glycerol, its advantage is that higher yields could be obtained due to the higher average degree of reduction of carbon atoms (κ=4.67) than with glucose (κ=4) (*100*). Consequently, by using glycerol, the yield of PA and recovery rate from glycerol will increase while acetic acid formation will decrease (100:1) (*14*, *30*). Therefore, reusing agro-industrial waste obtained from biodiesel can reduce the total cost of PA production up to 70% (*19*).

Coral *et al*. (*19*) used various carbon sources to test the effect of substrate on PA fermentation by 9 strains of *Propionibacterium*. Lactate showed the highest PA productivity and yield. Additionally, lactate enhanced the rate of PA production compared to molasses; it is not degraded *via* glycolytic route; therefore, acid biosynthesis is easier. Another advantage of lactate is that due to low pH variation during its degradation, there is no demand for constant control of pH, which is needed for glycerol and sugarcane molasses.

### Mixed carbon source

Although common practice for propionic acid fermentation is usually the use of a single carbon source, this is generally not enough for the growth of *Propionibacterium* and production of PA. Therefore, the application of mixed carbon sources could be proposed as an effective strategy for increasing PA production through kinetics alterations.

Jerusalem artichoke-based media, which contain different carbohydrates as mixed carbon sources, were used with the addition of 10 g/L yeast extract for the production of propionic acid by *P. acidipropionici*, with propionic acid concentration and productivity of 40 g/L and 0.26 g/(L·h), respectively (*88*). The mixture of glucose and glycerol yielded 29.2 g/L of propionic acid (*89*). The yield was quite low, and the used medium was relatively expensive. Co-fermentation of glucose and glycerol at a suitable mass ratio gave higher yield and concentration of propionic acid.

### Fermentation time

Each microorganism has a particular growth phase, which depends on the fermentation variables including pH, temperature, culture medium or desired properties of the final product. Fermentation time strongly depends on the growth rate of the microorganism. Selection of appropriate strains of *Propionibacterium* is also one of the most considerable factors. However, in propionic acid fermentation, its concentration could reach its maximum at a certain time of production; however, any prolongation of the bioprocess might cause a decrease in the final PA concentration. With prolongation of fermentation time, productivity is reduced due to the accumulation of inhibitory factors in the fermentor. Therefore, the optimization of fermentation duration is essential in order to obtain maximum productivity (*23*).

In conventional one-step batch fermentation, production period lasts up to 200 h; however, this period can be prolonged by using advanced bioreactor systems (*91*). By applying several repeated batch cycles with continuous recycle of cells, production time could be prolonged. During the cell-recycle fermentation for 11 consecutive batches the production time of PA by *P. acidipropionici* DSM 4900 lasted over 500 h (*101*). High concentration of PA could be produced by using the fed-batch system. The considerably high concentration of PA (71.8 g/L) was obtained after 12 days of fermentation of hemicellulose hydrolysate and corncob molasses using *P. acidipropionici* ATCC 4875 (*9*). Immobilization systems also alter PA production time radically. Immobilization of *P. acidipropionici* DSM 4900 in polyethylenimine-treated Luffa (PEI-Luffa) allowed a batch fermentation with a total production time of 225.5 h, which is considered longer than with free cells (126.75 h) (*91*). Recent studies have shown that the production of biofilm and exopolysaccharides (EPS) facilitates immobilization of *Propionibacterium freudenreichii* and *Propionibacterium acidipropionici*. The formation of biofilm and EPS can be induced by triggering factors such as NaCl and citric acid (*102*).

### Nitrogen source

*Propionibacterium* spp. can digest nitrogen sources including peptone, corn steep liquor and yeast extract, which can enhance the PA production (*16*). Production of PA on corn steep liquor as an agro-industrial effluent exhibits relatively high yield (0.79 g/g) and productivity (5.20 mg/(L·h) (*103*). Although the addition of different concentrations of nitrogen source in the range 5-40 g/L was reported in various studies, the most frequent were 5 and 10 g/L (*104*). However, more investigations to find low-cost nitrogen sources is recommended.

## APPLICATION OF PROPIONIC ACID

Propionic acid is beneficial to the human body and may play a role in satiety and energy homeostasis by specific mechanisms including activation of free fatty acid receptors, reducing lipogenesis level and glucose homeostasis (*105*). Small quantities of propionic acid as a commercial antimicrobial agent (E 280) are available in many foods such as dairy products (*106*). It can be applied to produce characteristic holes and nutty flavour in Swiss-type cheese (*81*).

**Fig. 1:**
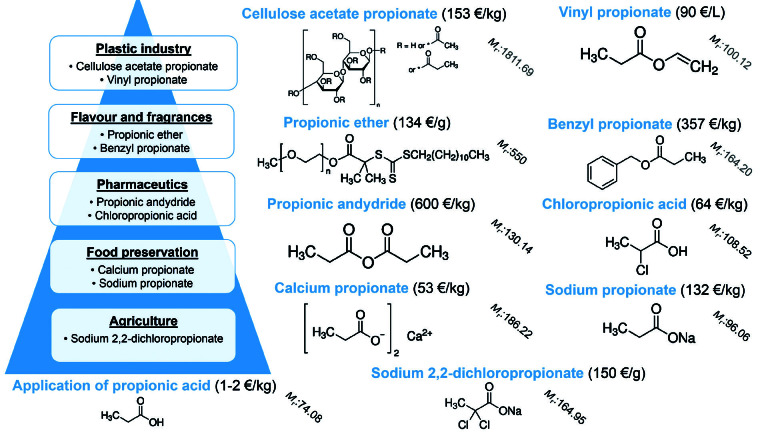
Chemical structure, relative molecular mass (*M*_r_) and approximate price (in €) of propionic acid derivatives in different fields of propionic acid application

The propionic acid in low concentration can slightly promote citric acid production (*107*) and in combination with acetic acid it improves hydrogen production rates during fermentation (*108*). It can be used for enzyme-catalyzed synthesis of esters from alcohols (*17*) as well as in P ethanol fermentation (*17*). Fig. 1 shows the properties and approximate price (in €) of propionic acid derivatives in different fields of propionic acid application.

As shown in Fig. 1, propionic acid is sold for about 1-2 €/kg for use in different industries including production of herbicides, pharmaceuticals, polymers (*e.g.* acrylonitrile cellulose fibre and modification of carbide slag) and perfumes (*57*, *109*, *110*).

### Antimicrobial agent

Numerous microorganisms can produce propionic acid *via* fermentation, while many of them can metabolize it. PA shows inhibitory effect against the microorganisms that metabolize it by accumulation in the cells, blocking metabolic pathways and consequently resulting in the inhibition of enzymes. Depending on the concentration, PA lowers the intracellular pH and inhibits microbial growth due to anion accumulation.

Propionic acid, as a relatively strong organic acid, has been employed as an antimicrobial agent in foodstuffs such as dairy and baking products, and in animal feed preservation. Instead of using antibiotics, which could lead to antibiotic resistance, feed can be treated with PA for its protection from bacterial and fungal degradation (*111*, *112*). PA is added to many poultry feed products to reduce the contamination by *Salmonella* spp. and undesired mould formation (*113*, *114*). In addition to antimicrobial activity, the PA in feed has shown to improve ruminal productivity by enhancing substrate degradation (8%) and reducing methane production (20%) (*115*, *116*). Unlike acetate, PA reduces the hydrogen transformation into methane (*117*). Application of lactic acid bacteria (LAB) can improve the production of PA by increasing the concentration of lactic acid and water-soluble carbohydrates in the rumen (*118*, *119*).

### Anti-inflammatory agent

Since the last century, there has been increasing need to discover novel anti-inflammatory agents with high efficiency for the treatment of many diseases. Several types of organic acids have been used for this purpose; however, as a prerequisite, in general, only nitrogen-free and non-steroidal compounds have been recognized as useful agents. Propionic acid, in its common chemical structure (C_3_H_6_O_2_), is free from nitrogen so it is widely used to produce anti-inflammatory agents (*120*, *121*).

Many different chemical groups could be added to PA to increase the anti-inflammatory effect. Studies confirmed that PA with an aryl group (profens *i.e*. 2-arylpropionic acid derivatives) is an important part of non-steroidal anti-inflammatory agents, which are widely prescribed against diseases such as arthritis and rheumatism (*122*).

Recently, new compounds have been introduced as possible additional agents with PA. Some propionic acid-based drugs used sas anti-inflammatory agents may have gastric ulcerogenic activity, which is an undesired effect for patients. 2-2-Fluoro-4-(2-oxocyclopentyl)methyl]phenyl}propionic acid can be incorporated in the formulation to eliminate this gastric effect (*123*).

### Herbicide

Wide ranges of herbicides have been utilized in modern agricultural methods in order to eliminate the target organisms. However, these herbicides may also affect the beneficial activities of non-target organisms that grow on the crops. Thus, it is essential to use biodegradable, target-specified agents such as derivatives of PA as promising herbicides to avoid agricultural expenses (*124*).

Far from other available artificial herbicides, propionic acid biodegrades firstly into acetic and formic acids, then to carbon dioxide and water; thus, it does not pose any threat to the environment. It is less caustic and corrosive than formic acid, another common herbicide. If proper formulation and respiratory protection are used, PA does not cause any health hazards during apllication. Propionic acid can control both monocotyledonous and dicotyledonous plants and it is an effective pre-emergent and post-emergent herbicide (*6*).

Some microorganism species are capable of degrading herbicides, specifically chiral forms of mecoprop ((RS)-2-(4-chloro-2-methylphenoxy)propionic acid) with different degradation rates. Previous investigations indicated that the use of a certain form of propionic acid-based herbicides decreases the degradation of mecoprop by these organisms, which, consequently, increases the effectiveness of the mentioned agents (*125*). However, after the application of herbicides, it is important to remove them from the applied region since they may be considered as a potential health hazard. In order to eliminate these agents, many microorganism species may be used efficiently (*126*).

### Preservative and safe food additive

Regarding the unstable physical conditions such as heat, excessive moisture, unpredictable rainfall, and also poor drying conditions, the addition of preservatives into food is very important since they tend to prevent the possible spoilage that could lead to food poisoning (*127*).

Propionic acid and its Ca, K and Na salts are common food additives used for food preservation. Wheat is usually cross-contaminated during harvest and especially in unfavourable storage conditions by fungi resulting in quality and economic losses. The use of PA and its salts may eliminate these contaminations during storage of crops (*20*).

Another method to increase the effect of PA as a food preservative is introducing this acid by specific carrier substances (*e.g.* vermiculite). The vermiculite pores of a certain diameter allow PA to penetrate particularly inside the grains.

Propionic acid is a generally recognised as safe (GRAS) food preservative. Some studies have reported that PA can exacerbate autism spectrum disorder (ASD) symptoms in humans. Besides *Propionibacterium*, gut bacteria produce PA by fermentation. As a result of *in vivo* production, PA can pass through the blood-brain and gut-blood barriers. Thereby, PA can cause neuroactive effects similar to ASD. Several cases of provoked ASD symptoms in children as a result of consumption of processed wheat or dairy products containing PA as food preservative have been reported (*128*, *129*).

## CONCLUSIONS

This article presents the aspects of propionic acid (PA) production by *Propionibacterium* sp. in the submerged system. Proper control of the substrate, culture conditions, type and bioreactor scale is important to ensure successful production of PA. The pH value and temperature are among the most important factors influencing the PA productivity. To determine substrate consumption rate, it is necessary to study the kinetics of *Propionibacterium* sp. The production of PA can be enhanced through the application of metabolically engineered mutants. Metabolic engineering should be studied as an essential tool to obtain better PA producers that show excellent resistance to acidic conditions, limited amount of substrates and are also easily adapted to different fermentation systems. Application of new immobilization techniques can be efficiently used with bioreactor systems and can bring signifcant economic advantage for PA production. All mentioned techniques should be investigated more to adapt to industrial production of PA.
